# Real -world experience with anti-obesity medications treatment in children and adolescents with overweight and obesity in Israel

**DOI:** 10.1038/s41366-025-01801-w

**Published:** 2025-05-15

**Authors:** Shlomit Shalitin, Moshe Phillip, Michal Yackobovitch-Gavan

**Affiliations:** 1 The Jesse Z. and Sara Lea Shafer Institute of Endocrinology and Diabetes, National Center for Childhood Diabetes Schneider Children’s Medical Center of Israel, Petah Tikva, Israel; 2 Faculty of Medical and Health Sciences, Tel Aviv University, Tel Aviv, Israel; 3 Dept. of Epidemiology and Preventive Medicine, School of Public Health, Faculty of Medicine, Tel Aviv University, Tel Aviv, Israel

**Keywords:** Obesity, Paediatrics

## Abstract

**Background:**

Childhood obesity is a major public health concern, associated with early-onset comorbidities and a high likelihood of persisting into adulthood. Anti-obesity medications (AOMs) may serve as an adjunct to lifestyle modifications for managing pediatric obesity.

**Objective:**

To evaluate prescribing patterns, weight outcomes, and cardiometabolic impacts of AOMs among children and adolescents aged 10–18 years within Clalit Health Services (CHS), the largest health maintenance organization in Israel.

**Subjects/Methods:**

This retrospective observational study analyzed data from CHS’s electronic database (2017–2024). The study cohort included 307 208 children with BMI measurements exceeding World Health Organization (WHO)-defined thresholds for overweight or obesity. Among these, 2236 (0.7%) were prescribed AOMs (metformin, GLP-1 receptor agonist, or orlistat). A secondary analysis assessed longitudinal changes in BMI z-scores and cardiometabolic parameters among individuals who purchased at list two prescriptions of AOMs.

**Results:**

AOMs prescriptions were more common among females, younger patients, those with higher BMI z-scores, and medium-to-high socioeconomic position (SEP) levels. Children prescribed AOMs exhibited a higher prevalence of obesity-related comorbidities and greater engagement with dietitians and endocrine specialists. Metformin was the most commonly prescribed medication (73.8%), followed by GLP-1 receptor agonist (24.5%) and orlistat (1.7%). Females demonstrated higher rates of medication adherence and longer treatment durations than males. Among the 1717 participants with ≥2 AOMs purchases, BMI z-scores significantly declined during treatment, accompanied by reductions in blood glucose, HbA1c, triglycerides, and total cholesterol, and increases in HDL cholesterol. BMI z-scores and cardiometabolic parameters partially regressed after treatment cessation but remained improved compared to baseline.

**Conclusions:**

AOMs demonstrate potential for weight management and cardiometabolic improvement in children with obesity, particularly among those with severe obesity and comorbidities, within real-world settings. However, the modest utilization rate highlights the need for improved accessibility and further real-world evidence to optimize treatment strategies for pediatric obesity.

## Introduction

Childhood obesity has reached pandemic proportions, presenting one of the most critical public health challenges of our era [1]. The underlying mechanisms driving excessive weight gain are complex, involving a dynamic interaction of genetic, biological, behavioral (e.g., sedentary lifestyles, prolonged screen exposure), and socioeconomic factors [2–4].

The increasing prevalence of childhood obesity has been associated with the early onset of comorbidities traditionally observed in adults, including hypertension, dyslipidemia, type 2 diabetes (T2D), obstructive sleep apnea (OSA), and non-alcoholic fatty liver disease (NAFLD) [5]. Furthermore, children with obesity face an increased risk of persisting into adulthood with obesity, a condition linked to greater morbidity and mortality risks [6]. As such, addressing the global childhood obesity epidemic has become an urgent public health priority.

Lifestyle interventions, encompassing dietary modifications and enhanced physical activity, are fundamental in the prevention and management of pediatric obesity.

In clinical pediatrics, a reduction in BMI z-score of 0.2–0.25 is recognized as clinically significant, as it correlates with improvements in cardiovascular and metabolic risk factors [7]. This change is roughly equivalent to a 5% decrease in BMI, which is associated with notable improvements in obesity-related comorbidities and mortality risk [8].

Despite its importance, lifestyle modification interventions typically yield only modest reductions in weight among children and adolescents with overweight or obesity [9]. Pharmacological therapy, regarded as a secondary approach, is generally reserved for individuals aged 10 years and older with severe obesity who have not achieved sufficient weight reduction through lifestyle interventions. This strategy is particularly applicable to those with obesity-related comorbidities, such as impaired glucose tolerance (IGT), insulin resistance, steatohepatitis, or a significant family history of cardiometabolic conditions [10].

A range of anti-obesity medications (AOMs) with diverse mechanisms of action have received approval for pediatric use from regulatory bodies, including the U.S. Food and Drug Administration (FDA) and the European Medicines Agency (EMA) [11]. Despite experimental evidence supporting the efficacy of these AOMs, it is important to validate it with information from real-world studies. Unfortunately, real-world data concerning AOMs utilization, effects on weight outcomes, and impact on cardiometabolic risk factors in the pediatric population remain limited [12–15].

The aim of this study was to examine current prescribing practices of AOMs among the pediatric population with overweight and obesity within Clalit Health Services (CHS) and to evaluate the impact of treatment with these medications on weight status and cardiometabolic outcomes.

## Subjects and methods

### Data source

This observational retrospective study was conducted using the electronic database of CHS, an Israeli payer-provider integrated health care system that serves about 54% of the Israeli population. The database is accumulated by continuous real-time input from physicians and health service providers, and includes patient demographic, socioeconomic, and clinical characteristics, hospital discharge and outpatient clinic diagnoses, laboratory test results, medical treatments, and medication dispensation information. Data were extracted from CHS using the Clalit Research Data sharing platform powered by MDClone (https://www.mdclone.com).

### Study population

The study population comprised children and adolescents aged 10–18 years with at least one recorded BMI measurement between January 2017 and December 2024, meeting the World Health Organization (WHO) criteria for overweight (defined as the 85th ≤ BMI ≤97th percentile [1 ≤ BMI-Z score ≤2]) or obesity (defined as BMI >97th percentile [BMI-Z score >2]) [16]. Individuals with implausible measurements, defined as BMI > 80 kg/m², were excluded from the analysis.

Between 2017 and 2024, the primary pharmacological treatments for pediatric obesity available in Israel comprised orlistat, a pancreatic lipase inhibitor that decreases fat absorption in the intestine [17]; liraglutide 3 mg, a glucagon-like peptide-1 (GLP-1) receptor agonist that modulates appetite and delays gastric emptying [18]; and metformin, an oral antidiabetic agent that reduces hepatic glucose production and enhances peripheral glucose uptake in muscle tissue [19].

The initiation and continuation of AOMs in Israel necessitate concurrent lifestyle modifications and regular follow-ups with a dietitian. Clinical guidelines recommend evaluating the efficacy of AOMs after three months of treatment and discontinuing them if they prove ineffective for the patient.

Orlistat and metformin are included in the national healthcare services basket and are available at a low cost to patients who meet the clinical eligibility criteria. Liraglutide 3 mg, on the other hand, is covered under supplemental health insurance plans, which are accessible to approximately 73% of CHS members. This medication is prescribed under specific conditions, including appropriate BMI thresholds, the presence of obesity-related comorbidities, and supervision by a dietitian, although it requires a higher copayment.

The study compared children and adolescents prescribed one or more AOMs with those who did not receive AOMs prescriptions.

A secondary analysis focused exclusively on individuals who purchased at list two prescriptions of AOMs, evaluating longitudinal changes in BMI and cardiometabolic outcomes.

Demographic parameters, including sex, ethnicity, age, and socioeconomic position (SEP), were extracted from medical records. Data collected during the study period encompassed anthropometric measurements (height, weight, and calculated BMI), and the presence of obesity-related comorbidities. These comorbidities included dyslipidemia, T2D, IGT, hypertension, OSA, NAFLD, and pseudotumor cerebri (PTC), as well as polycystic ovary syndrome (PCOS) in adolescent girls. Additional data were obtained on screening practices during the study period, including assessments of fasting blood glucose, HbA1c, lipid profiles, and liver enzymes.

Follow-up data included repeated weight and height measurements, referrals to dietitians or endocrine specialists, and prescriptions and purchases of AOMs, including orlistat, GLP-1 receptor agonist, and metformin. To assess compliance with AOMs treatment in this retrospective study, at least two documented dispensing events were required.

For individuals receiving AOMs, data included last measurement before the first AOMs purchase, nadir (lowest measurement recorded during medication use), last measurement before the end of AOMs treatment and the first measurement after the end of AOMs treatment. Weight loss outcomes were expressed as changes in BMI-Z scores. Additionally, data on changes in laboratory parameters, including fasting blood glucose, HbA1c, lipid profiles, and liver enzymes, were collected during the course of AOMs treatment.

The SEP index of the Israel Central Bureau of Statistics classifies and characterizes statistical areas within municipalities and local councils based on the socioeconomic status of their populations. This index is derived from an adjusted calculation of 14 variables that assess social and economic levels in the domains of demographics, education, standard of living, and employment. The SEP classification for a given residence is determined according to the socioeconomic level of the population within towns and neighborhoods, as evaluated in 2015 [20]. The SEP clusters are scored on a scale from 1 to 10, with 1 indicating the lowest SEP and 10 representing the highest. For the purposes of this study, these 10 clusters were categorized into three groups: low SEP (clusters 1-4), medium SEP (clusters 5–6) and high SEP (clusters 7–10).

BMI was calculated as weight (in kilograms) divided by height (in meters) squared. To compare BMI values across age groups by sex, BMI-Z scores were calculated using the growth chart percentiles of the WHO [16].

The study was approved by the local institutional ethics committee of Rabin Medical center (RMC- 0222-24) in keeping with the principles of the Declaration of Helsinki. Written subject informed consent was not required due to the retrospective design and the anonymous collection of the data.

### Statistical analysis

Statistical analyses were performed using SPSS software, version 29 (SPSS, Inc., Chicago, Illinois). Data are presented as *n* (%) or median (interquartile range for skewed distributions).

Comparisons between groups and between sexes were conducted using Pearsons’ chi square tests for categorial variables or Mann–Whitney U tests for numerical variables.

Mixed models repeated measures analysis were conducted to evaluate the changes in BMI measurements and laboratory results in children treated AOMs (all AOMs and by medication type) among four time points: last measurement before the first AOMs purchase, minimal measurement during AOMs treatment, last measurement before the end of AOMs treatment and the first measurement after the end of AOMs treatment. The models were specified with a within-group factor of time (the 4 time points), a between-group factor of sex, and the interaction of sex with time. The data are expressed as estimated marginal means and standard errors.

## Results

**Fig. 1:**
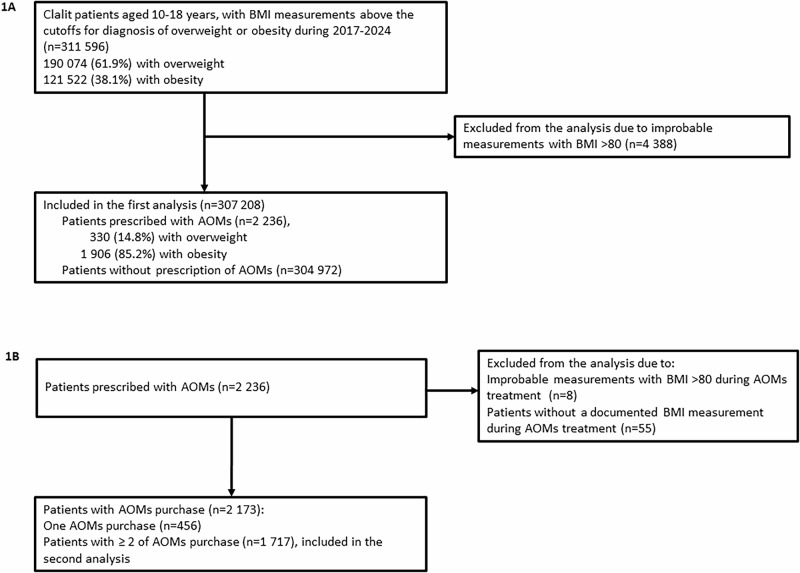
Flow chart of patients with overweight and obesity included in the study cohort. **A** patients included in the initial analysis of the study cohort; those prescribed anti-obesity medications (AOMs) and those without prescription of AOMs **B** patients prescribed anti-obesity medications (AOMs) included in the secondary analysis.

During the study period, a total of 311,596 children aged 10–18 years had BMI measurements exceeding the WHO-defined thresholds for overweight or obesity, with 61.9% classified with overweight and 38.1% with obesity. Following the exclusion of individuals with implausible BMI values (>80 kg/m², *n* = 4 388), the final cohort comprised 307 208 children. Among these, 2236 individuals (0.7%) were prescribed AOMs, including 1906 (85.2%) children with obesity and 330 (14.8%) children with overweight (Fig. 1A).

Table 1 presents the characteristics of the study cohort, stratified by AOMs prescription status. Children who received AOMs prescriptions exhibited distinct demographic and clinical differences compared to those who did not. Specifically, a significantly higher proportion of females were prescribed AOMs (59.2% vs. 46.8%, *p* < 0.001). Additionally, the prevalence of Jewish ethnicity was greater among those prescribed AOMs compared to non-recipients (68.7% vs. 65.0%, *p* = 0.02 for males; 73.1% vs. 67.2%, *p* < 0.001 for females). Children prescribed AOMs were also younger at the time of their first BMI measurement (within the 10–18-year age range) compared to those not prescribed AOMs (median age: 11.9 vs. 13.1 years for males, and 12.3 vs. 13.1 years for females; *p* < 0.001 for both sexes). Furthermore, they had significantly higher BMI z-scores at first measurement (median BMI-SDS: 3.05 vs. 1.8 for males, and 2.8 vs. 1.69 for females; *p* < 0.001 for both sexes) and a greater prevalence of obesity (88% vs. 40.7% for males and 83.4% vs. 34.5% for females; *p* < 0.001 for both sexes). Among females, those prescribed AOMs were less frequently represented in lower socioeconomic position (SEP) categories compared to their counterparts who were not prescribed AOMs (24.3% vs. 29.7%, *p* < 0.001).

**Table 1:** Characteristics of the study cohort according to prescription of anti- obesity medications (AOMs).

	Patients with prescription (≥1) of AOMs before age 18 years (*n* = 2236)	Patients without prescription of AOMs before age 18 years (*n* = 304,972)	P1
**Sex**			
**males**	913 (40.8%)	16,2351 (53.2%)	<0.001
**females**	1323 (59.2%)	14,2621 (46.8%)	
**Ethnicity** ***n*** **(%)**			
**males**			
**Jews**	627 (68.7%)	10,5536 (65.0%)	
**Arabs**	286 (31.3%)	56,815 (35.0%)	0.020
**females**			
**Jews**	967 (73.1%)	95,895 (67.2%)	
**Arabs**	356 (26.9%)	46,726 (32.8%)	<0.001
**P2**	0.023	<0.001	
**Age at first BMI measurement (between age 10–18 years) Median (IQR)**			
**males**	11.9 (10.7, 13.4)	13.1 (11.3, 14.8)	<0.001
**females**	12.3 (10.9, 13.7)	13.1 (11.3, 14.8)	<0.001
**P2**	<0.001	0.499	
**BMI-Z score (at first measurement)**			
**males**	3.05 (2.50, 3.52)	1.80 (1.36, 2.40)	<0.001
**females**	2.80 (2.27, 3.26)	1.69 (1.32, 2.23)	<0.001
**P2**	<0.001	<0.001	
**Weight status categories** ***n*** **(%)**			
**Males**			
**Overweight**	110 (12.0%)	96,298 (59.3%)	
**Obesity**	803 (88.0%)	66,053 (40.7%)	<0.001
**Females**			
**Overweight**	220 (16.6%)	93,426 (65.5%)	
**Obesity**	1 103 (83.4%)	49,195(34.5%)	<0.001
**P2**	0.003	<0.001	
**SEP level** ***n*** **(%) males (the word males has to be in a separate row below)**			
**Low**	249 (29.6%)	46,423 (30.8%)	
**Medium**	480 (57.0%)	81,366 (54.1%)	0.168
**High**	112 (13.3%)	22,715 (15.1%)	
**missing**	72	11,847	
**females**			
**Low**	301 (24.3%)	39,254 (29.7%)	
**Medium**	746 (60.3%)	72,507 (54.9%)	<0.001
**High**	191 (15.4%)	20,295 (15.4%)	
**missing**	85	10,565	
**P2**	0.021	<0.001	

The data are presented as *n* (%) or median (interquartile range, skewed distribution). Weight status category according the WHO criteria, P1 represent comparison between groups and P2 represent comparison between sexes using Pearsons’ chi square tests for categorial variables or Mann–Whitney U tests for numerical variables. SEP, Socioeconomic Position.

Within the group prescribed AOMs, males of Arab ethnicity, were significantly more prevalent than their female counterparts (*p* = 0.023). Additionally, males prescribed AOMs were significantly younger at the time of first prescription (*p* < 0.001), had higher BMI z-scores (*p* < 0.001), and a greater proportion were classified as having obesity (*p* = 0.003) compared to females. In contrast, a higher proportion of females prescribed AOMs belonged to medium and high SEP levels compared to their male counterparts (*p* = 0.021).

Table 2 details obesity-related comorbidities, referrals to dietitians, and endocrine evaluations, stratified by AOMs prescription status. Children receiving AOMs had significantly higher rates of all obesity-related comorbidities, including dyslipidemia, T2D, IGT, hypertension, OSA, NAFLD, PTC, and PCOS in females (*p* < 0.001 for all). Furthermore, those receiving AOMs were referred to dietitians (*p* < 0.001) and for endocrine evaluations (*p* < 0.001) at significantly higher rates compared to non-recipients. Among those prescribed AOMs, males exhibited significantly higher prevalence of hypertension (*p* = 0.003) and NAFLD (*p* = 0.005) compared to females. However, a lower percentage of males compared to females were referred for dietitian consultations (*p* = 0.025).

**Table 2:** Documented obesity-related comorbidities, referral to dietician or endocrine evaluation according to prescription of anti-obesity medications (AOMs).

	Patients with prescription (≥1) of AOMs at age < 18 years	Patients without prescription AOMs at age < 18 years	P1
***n***			
**Males**	913	16,2351	
**Females**	1 323	14,2621	
**Dyslipidemia**			
**males**	93 (10.2%)	2274 (1.4%)	<0.001
**females**	138 (10.4%)	2347 (1.6%)	<0.001
**P2**	0.852	<0.001	
**Type 2 diabetes**			
**males**	54 (5.9%)	56 (0.03%)	<0.001
**females**	78 (5.9%)	83 (0.06%)	<0.001
**P2**	0.985	0.002	
**IGT**			
**males**	101 (11.1%)	841 (0.5%)	<0.001
**females**	126 (9.5%)	934 (0.7%)	<0.001
**P2**	0.236	<0.001	
**Hypertension**			
**males**	94 (10.3%)	1885 (1.2%)	<0.001
**females**	90 (6.8%)	939 (0.7%)	<0.001
**P2**	0.003	< 0.001	
**OSA**			
**males**	19 (2.1%)	209 (0.1%)	<0.001
**females**	19 (1.4%)	197 (0.1%)	<0.001
**P2**	0.246	0.478	
**NAFLD**			
**males**	263 (28.8%)	2926 (1.8%)	<0.001
**females**	311 (23.5%)	2109 (1.5%)	<0.001
**P2**	0.005	<0.001	
**PTC**			
**males**	16 (1.8%)	149 (0.1%)	<0.001
**females**	34 (2.6%)	484 (0.3%)	<0.001
**P2**	0.199	<0.001	
**PCO**			
**females**	21 (1.6%)	100 (0.1%)	<0.001
**Referral to dietician**			
**males**	647 (70.9%)	25,127 (15.5%)	<0.001
**females**	994 (75.1%)	35,003 (24.5%)	<0.001
**P2**	0.025	<0.001	
**Referral to endocrine evaluation**			
**males**	510 (55.9%)	11,190 (6.9%)	<0.001
**females**	778 (58.8%)	13,323 (9.3%)	<0.001
**P2**	0.166	<0.001	

Data is presented as *n* (%). P1 represent comparison between groups using Pearsons’ chi square tests. P2 represent comparison between sexes using Pearsons’ chi square tests.

*IGT* impaired glucose tolerance, *OSA* obstructive sleep apnea, *NAFLD* non-alcoholic fatty liver disease, *PTC* pseudotumor cerebri, *PCO* polycystic ovary.

Of the 2236 children prescribed AOMs, exclusions were made for those lacking a documented BMI measurement during AOMs treatment (*n* = 55) and those with implausible BMI measurements during AOMs treatment (BMI > 80 kg/m², *n* = 8). Following these exclusions, the final cohort comprised 2173 patients prescribed AOMs, of whom 1717 (79%) had two or more documented medication purchases (Fig. 1B).

Table 3 compares patients who made a single AOMs purchase to those with two or more purchases. Individuals with ≥2 AOMs purchases demonstrated a significant predominance of females (*p* = 0.020), and higher BMI z-scores before the time of the first purchase (*p* = 0.046). Additionally, this group had a significantly greater prevalence of specific obesity-related comorbidities, including T2D (*p* < 0.001), hypertension (*p* = 0.047), and NAFLD (*p* = 0.018). This group also exhibited a higher referral rate for dietitian consultations (*p* = 0.031).

**Table 3:** Comparison between patients with only one of anti-obesity medications (AOMs) purchase and those with ≥ 2 purchases.

	One AOMs purchase (*n* = 456)	≥ 2 of AOMs purchase (*n* = 1 717)	*P*
**Sex males** ***n*** **(%)**	206 (45.2%)	672 (39.1%)	0.020
**Ethnicity** ***n*** **(%)**			
**Jews**	315 (69.1%)	1240 (72.2%)	
**Arabs**	141 (30.9%)	477 (27.8%)	0.187
**Age at first purchase, years**			
**Median (IQR)**	15.2 (13.1, 16.5)	15.0 (13.3, 16.5)	0.595
**BMI-Z score (before first purchase)**			
**Median (IQR)**	2.96 (2.08, 3.53)	3.03 (2.37, 3.52)	0.046
**SEP level** ***n*** **(%)**			
**Low**	126 (28.9%)	410 (25.5%)	
**Medium**	257 (58.9%)	948 (59.1%)	0.142
**High**	53 (12.2%)	247 (15.4%)	
**missing**	20	112	
**Dyslipidemia** ***n*** **(%)**	43 (9.4%)	185 (10.8%)	0.405
**Type 2 diabetes**	9 (2.0%)	122 (7.1%)	<0.001
**IGT**	44 (9.7%)	180 (10.5%)	0.602
**Hypertension**	27 (5.9%)	151 (8.8%)	0.047
**OSA**	6 (1.3%)	32 (1.9%)	0.428
**NAFLD**	99 (21.7%)	467 (27.2%)	0.018
**PTC**	9 (2.0%)	39 (2.3%)	0.701
**PCO females**	2 (0.4%)	18 (1.0%)	0.226
**Referral to dietician**	321 (70.4%)	1294 (75.4%)	0.031
**Referral to endocrine evaluation**	258 (56.6%)	1011 (58.8%)	0.375

Data is presented as *n* (%). *P* represent comparison between groups using Pearsons’ chi square tests.

*IGT* impaired glucose tolerance, *OSA* obstructive sleep apnea, *NAFLD* non-alcoholic fatty liver disease, *PTC* pseudotumor cerebri, *PCO* polycystic ovary.

Among those with only one AOMs purchase, 2.6% (9 out of 350) of those who purchased metformin had also a documented diagnosis of T2D and among those with two or more purchases, 9.6% (122 out of 1267) of patients who purchased metformin had also a T2D diagnosis.

Table 4 presents the characteristics of patients with ≥2 AOMs purchases, stratified by medication type and sex. The most frequently purchased medication was metformin (73.8%), followed by GLP-1 receptor agonist (Liraglutide) (24.5%) and Orlistat (1.7%). No significant differences were observed in the distribution of prescribed medications between sexes. However, females were prescribed GLP-1 receptor agonist at a significantly older age compared to males (*p* = 0.006).

**Table 4:** Characteristics of patients with ≥ 2 purchases of anti-obesity medications (AOMs) according to medication type: comparison between males and females.

	Males *n* = 672 (39.1%)	Females *n* = 1 045 (60.9%)	*P*
**Medication type (at first medication purchase)** ***n*** **(%)**			
**Orlistat**	8 (1.2%)	22 (2.1%)	0.072
**Metformin**	483 (71.9%)	784 (75.0%)	
**GLP1 receptor agonist**	181 (26.9%)	239 (22.9%)	
**Age, years (at first medication purchase) Median (IQR)**			
**Orlistat**	16.4 (14.3, 17.3)	15.7 (14.6, 17.2)	0.716
**Metformin**	14.6 (13.0, 16.2)	14.9 (13.2, 16.4)	0.159
**GLP1 receptor agonist**	16.2 (14.6, 17.8)	16.6 (14.7, 18.3)	0.006
**BMI-Z score (before first medication purchase) Median (IQR)**			
**Orlistat**	3.14 (1.57, 3.70)	2.73 (2.20, 3.40)	0.667
**Metformin**	3.02 (2.39, 3.54)	2.88 (2.08, 3.42)	0.006
**GLP1 receptor agonist**	3.36 (2.95, 3.70)	3.13 (2.58, 3.55)	<0.001
**Number of purchases Median (IQR)**			
**Orlistat**	2 (2,6)	3 (2, 5)	0.561
**Metformin**	7 (4, 16)	8 (4, 17)	0.232
**GLP1 receptor agonist**	5 (3, 10)	5 (3, 11)	0.893
**Treatment period (years) Median (IQR)**			
**Orlistat**	0.55 (0.29, 0.77)	0.60 (0.17, 1.25)	0.860
**Metformin**	1.51 (0.52, 3.38)	1.67 (0.60, 3.69)	0.159
**GLP1 receptor agonist**	0.55 (0.33, 1.14)	0.64 (0.32, 1.15)	0.520

The data are presented as *n* (%) or median (interquartile range, skewed distribution). *P* represent comparison between sexes using Pearsons’ chi square tests for categorial variables or Mann–Whitney U tests for numerical variables.

BMI z-scores before the time of the first medication purchase were significantly higher in males than in females for both metformin (*p* = 0.006) and GLP-1 receptor agonist (*p* < 0.001).

The median duration of treatment with all medication types was similar for males and females. Specifically, the median treatment duration for orlistat was 0.55 years in males and 0.6 years in females, for metformin, 1.51 years in males and 1.67 years in females, and for GLP-1 receptor agonist, 0.55 years in males and 0.64 years in females.

Table 5 summarizes the changes in BMI-Z score and laboratory parameters among children treated with AOMs, both overall and stratified by medication type. In both sexes, the initiation of AOMs therapy was associated with a significant reduction in BMI z-scores. Following treatment discontinuation, BMI z-scores increased but remained lower than baseline levels (P_time_<0.001). BMI-z scores were constantly higher in males compared to females (P_sex_<0.001), though similar pattern of change over time in males and females (P_time×sex_ = 0.987). Comparable trends for the over-time changes in BMI-z score were obtained for metformin and GLP-1 receptor agonist treatment. However, for Orlistat treatment, the changes in BMI-z score and other laboratory parameters did not reach statistical significance, likely due to the small sample size. Among those treated with AOMs, 55.1% achieved a maximal BMI-z score reduction of >0.2 SD during treatment, with no significant differences among medication types (Metformin 53.9%, GLP-1 receptor agonist 57.3%, Orlistat 58.3%, *P* = 0.588).

**Table 5:** Changes in BMI measurements and laboratory results in children treated with anti-obesity medications (AOMs)- all AOMs and by medication type.

	Last measurement before first drug purchase	Minimal measurement during treatment period*	Last measurement before end of treatment	First measurement after end of treatment	*P* time	*P* sex	P time×sex
**All AOMs**							
**BMI-Z Score**							
**Males**	3.15 (0.05) ^a^	2.63 (0.04) ^b^	2.93 (0.04) ^c^	2.87 (0.06) ^c^	<0.001	<0.001	0.987
**Females**	2.98 (0.05) ^a^	2.47 (0.03) ^b^	2.76 (0.03) ^c^	2.73 (0.04) ^c^	<0.001		
**Fasting blood glucose(mg/dL)**							
**Males**	140.8 (3.3) ^a^	96.4 (2.8) ^b^	126.4 (4.2) ^c^	115.8 (3.5) ^d^	<0.001	<0.001	0.384
**Females**	128.6 (2.6) ^a^	92.2 (1.8) ^b^	115.6 (2.8) ^c^	109.2 (2.3) ^c^	<0.001		
**HbA1c (%)**							
**Males**	6.8 (0.10) ^a^	5.8 (0.08) ^b^	6.3 (0.08) ^c^	6.5 (0.16) ^a,c^	<0.001	<0.001	0.266
**Females**	6.4 (0.07) ^a^	5.6 (0.05) ^b^	5.9 (0.05) ^c^	6.0 (0.10) ^c^	<0.001		
**Triglycerides (mg/dL)**							
**Males**	153.7 (5.7) ^a^	99.9 (4.4) ^b^	141.7 (8.1) ^a^	141.8 (8.0) ^a^	<0.001	0.745	0.511
**Females**	152.5 (4.2) ^a^	106.9 (3.2) ^b^	135.6 (5.8) ^c^	135.7 (5.9) ^c^	<0.001		
**HDL- Cholesterol (mg/dL)**							
**Males**	42.0 (0.6) ^a^	48.0 (0.5) ^b^	42.2 (0.7) ^a,c^	43.8 (0.5) ^c^	<0.001	0.686	0.227
**Females**	42.1 (0.5) ^a^	47.0 (0.4) ^b^	43.2 (0.5) ^a,c^	44.2 (0.4) ^c^	<0.001		
**LDL-cholesterol (mg/dL)**							
**Males**	96.3 (1.4)	92.1 (2.0)	95.3 (1.6)	93.3 (2.0)	0.298	0.659	0.758
**Females**	97.9 (1.3)	93.3 (1.6)	93.9 (1.5)	94.2 (1.6)	0.065		
**Total cholesterol (mg/dL)**							
**Males**	167.6 (1.9) ^a^	150.8 (1.5) ^b^	164.3 (2.1) ^a^	165.9 (2.6) ^a^	<0.001	0.321	0.996
**Females**	168.6 (1.4) ^a^	152.5 (1.1) ^b^	165.4 (1.6) ^a^	167.3 (2.0) ^a^	<0.001		
**ALT (U/L)**							
**Males**	33.0 (1.4)	30.0 (1.8)	29.5 (1.5)	29.7 (1.9)	0.286	0.012	0.996
**Females**	36.0 (1.2)	33.1 (1.5)	31.9 (1.4)	32.3 (1.6)	0.087		
**Metformin (*n*** = **1 267)**							
**BMI-Z Score**							
**Males**	2.98 (0.07) ^a^	2.46 (0.05) ^b^	2.81 (0.05) ^c^	2.68 (0.07) ^c^	<0.001	0.002	0.965
**Females**	2.83 (0.06) ^a^	2.34 (0.04) ^b^	2.68 (0.04) ^c^	2.59 (0.05) ^c^	<0.001		
**Fasting blood glucose(mg/dL)**							
**Males**	153.4 (4.1) ^a^	101.0 (3.6) ^b^	135.2 (5.0) ^c^	126.1 (4.5) ^c^	<0.001	<0.001	0.322
**Females**	136.4 (2.7) ^a^	95.1 (2.3) ^b^	121.7 (3.3) ^c^	115.6 (2.8) ^c^	<0.001		
**HbA1c (%)**							
**Males**	6.7 (0.13) ^a^	5.8 (0.10) ^b^	6.3 (0.10) ^c^	6.5 (0.20) ^ac^	<0.001	<0.001	0.385
**Females**	6.5 (0.08) ^a^	5.6 (0.06) ^b^	5.9 (0.06) ^c^	5.9 (0.11) ^c^	<0.001		
**Triglycerides (mg/dL)**							
**Males**	159.6 (7.6) ^a^	101.6 (5.8) ^b^	144.1 (11.0) ^a^	144.4 (11.2) ^a^	<0.001	0.413	0.614
**Females**	156.3 (5.2) ^a^	106.8 (4.0) ^b^	134.2 (7.1) ^c^	134.7 (7.3) ^c^	<0.001		
**HDL- Cholesterol (mg/dL)**							
**Males**	41.5 (0.7) ^a^	47.0 (0.5) ^b^	42.2 (0.8) ^a^	43.0 (0.6) ^a^	<0.001	0.125	0.438
**Females**	42.1 (0.5) ^a^	46.7 (0.4) ^b^	43.4 (0.6) ^a,c^	44.1 (0.5) ^c^	<0.001		
**LDL-cholesterol (mg/dL)**							
**Males**	94.9 (1.7)	92.8 (2.6)	94.5 (1.9)	93.9 (2.6)	0.920	0.539	0.735
**Females**	98.2 (1.5)	93.1 (1.9)	94.3 (1.7)	94.1 (1.9)	0.114		
**Total cholesterol (mg/dL)**							
**Males**	166.4 (2.4) ^a^	150.7 (1.8) ^b^	164.0 (3.5) ^a^	164.3 (3.5) ^a^	<0.001	0.178	0.987
**Females**	169.0 (1.6) ^a^	152.1 (1.2) ^b^	165.6 (1.9) ^a^	166.9 (2.2) ^a^	<0.001		
**ALT (U/L)**							
**Males**	32.2 (1.6)	30.1 (2.2)	27.9 (1.8)	29.4 (2.2)	0.336	0.008	0.999
**Females**	35.9 (1.4)	33.3 (1.7)	31.4 (1.6)	33.1 (1.9)	0.190		
**GLP-1 receptor agonist (*n*** = **420)**							
**BMI-Z Score**							
**Males**	3.45 (0.05) ^a^	3.08 (0.05) ^b^	3.22 (0.05) ^c^	3.31 (0.06) ^ac^	<0.001	<0.001	0.978
**Females**	3.28 (0.05) ^a^	2.95 (0.05) ^b^	3.06 (0.05) ^c^	3.16 (0.06) ^ac^	<0.001		
**Fasting blood glucose(mg/dL)**							
**Males**	92.8 (1.3) ^a^	82.4 (1.0) ^b^	86.8 (1.7) ^c^	87.4 (1.2) ^c^	<0.001	0.876	0.972
**Females**	93.1 (1.3) ^a^	82.2 (1.2) ^b^	86.4 (2.1) ^b,c^	88.4 (1.5) ^c^	<0.001		
**HbA1c (%)**							
**Males**	6.9 (0.17) ^a^	5.7 (0.14) ^b^	6.2 (0.14) ^c^	6.5 (0.24) ^a,c^	<0.001	0.010	0.254
**Females**	6.3 (0.14) ^a^	5.6 (0.11) ^b^	5.9 (0.10) ^c^	6.4 (0.21) ^a^	<0.001		
**Triglycerides (mg/dL)**							
**Males**	136.0 (7.6) ^a^	95.8 (6.1) ^b^	135.2 (10.0) ^a^	135.3 (10.1) ^a^	<0.001	0.123	0.983
**Females**	144.0 (6.3) ^a^	107.8 (4.9) ^b^	142.2 (9.0) ^a^	142.9 (9.1) ^a^	<0.001		
**HDL- Cholesterol (mg/dL)**							
**Males**	42.8 (1.2) ^a^	50.4 (0.9) ^b^	42.4 (1.4) ^a^	45.5 (1.0) ^a^	<0.001	0.181	0.768
**Females**	41.8 (1.1) ^a^	48.2 (0.8) ^b^	42.4 (1.2) ^a^	44.5 (1.0) ^a^	<0.001		
**LDL-cholesterol (mg/dL)**							
**Males**	99.6 (2.3)	92.1 (3.1)	98.4 (2.8)	93.3 (3.1)	0.154	0.861	0.464
**Females**	97.1 (2.7)	95.5 (3.6)	93.2 (3.2)	96.0 (3.6)	0.818		
**Total cholesterol (mg/dL)**							
**Males**	170.1 (2.9) ^a^	151.5 (2.3) ^b^	166.5 (3.5) ^a^	168.3 (3.8) ^a^	<0.001	0.812	0.803
**Females**	167.8 (3.1) ^a^	154.3 (2.4) ^b^	165.9 (3.6) ^a^	170.6 (4.5) ^a^	<0.001		
**ALT (U/L)**							
**Males**	35.2 (2.8)	30.0 (3.5)	33.9 (3.2)	30.1 (3.5)	0.549	0.684	0.952
**Females**	37.3 (2.5)	32.0 (3.2)	33.0 (2.9)	30.5 (3.5)	0.355		
**Orlistat (*n*** = **30)**							
**BMI-Z Score**							
**Males**	3.37 (0.57)	2.63 (0.45)	3.24 (0.45)	2.31 (0.52)	0.452	0.023	0.402
**Females**	2.42 (0.37)	1.93 (0.21)	2.32 (0.21)	2.48 (0.26)	0.323		
**Fasting blood glucose(mg/dL)**							
**Males**	88.0 (4.4)	82.0 (3.1)	91.0 (5.0)	90.9 (3.3)	0.232	0.249	0.848
**Females**	85.2 (3.7)	81.1 (2.5)	82.7 (4.5)	89.3 (2.8)	0.187		
**HbA1c (%)**							
**Males**	7.7 (1.1)	5.6 (0.9)	5.9 (0.9)	7.5 (1.6)	0.424	0.043	0.354
**Females**	6.1 (0.4)	5.6 (0.4)	5.6 (0.3)	5.3 (0.6)	0.692		
**Triglycerides (mg/dL)**							
**Males**	163.8 (41.2)	99.6 (38.1)	162.7 (50.0)	162.8 (50.4)	0.612	0.014	0.402
**Females**	107.2 (15.4)	99.9 (12.2)	85.0 (21.2)	85.5 (21.8)	0.780		
**HDL- Cholesterol (mg/dL)**							
**Males**	46.5 (5.2)	47.0 (4.8)	32.5 (9.1)	43.3 (6.4)	0.547	0.411	0.657
**Females**	45.1 (3.1)	47.6 (2.5)	44.0 (4.0)	43.5 (3.1)	0.740		
**LDL-cholesterol (mg/dL)**							
**Males**	98.8 (13.8)	81.4 (15.1)	68.5 (23.9)	81.4 (15.1)	0.669	0.886	0.839
**Females**	100.2 (7.7)	74.8 (10.4)	85.6 (9.4)	74.8 (10.4)	0.164		
**Total cholesterol (mg/dL)**							
**Males**	171.4 (17.2)	145.0 (16.0)	118.0 (29.9)	174.7 (21.1)	0.336	0.721	0.338
**Females**	161.0 (8.2)	143.3 (6.6)	147.5 (10.3)	142.8 (12.2)	0.380		
**ALT (U/L)**							
**Males**	26.3 (7.6)	28.8 (8.3)	20.5 (13.1)	30.3 (9.3)	0.934	0.530	0.740
**Females**	29.2 (6.7)	37.1 (9.1)	36.3 (8.1)	21.6 (10.8)	0.643		

*For HDL-cholesterol—maximal measurement during treatment period Mixed models repeated measures analysis. Values are presented as estimated means and standard error (SE). Variables with different superscripts (a, b, c, d) significantly differ from each other at *p* < 0.05 at post hoc least significant difference pairwise comparisons.

AOMs initiation also resulted in significant reductions in blood glucose levels, HbA1c, triglycerides, and total cholesterol, alongside a significant increase in HDL cholesterol in both sexes. Post-treatment cessation, there was a trend toward rising levels of blood glucose, HbA1c, triglycerides, and total cholesterol, though levels remained below their pre-treatment values (P_time_<0.001). HDL cholesterol exhibited a decline after treatment cessation but remained elevated compared to baseline levels (P_time_<0.001).

All patterns of change of over-time in laboratory parameters were similar in males and females (all P_time×sex_ > 0.05).

## Discussion

Our study reveals that AOMs are prescribed to a relatively small proportion (0.7%) of children aged 10–18 years with overweight or obesity. This prevalence is significantly lower than the reported 8% prescription rate from a national cohort of children in the United States [15]. Additionally, children in our cohort who were prescribed AOMs had higher BMI z-scores compared to those reported by Czepiel et al. [14], based on data from a large unified healthcare system comprising two academic medical centers and three community teaching hospitals in the United States.

These findings may reflect differences in prescribing policies for AOMs among children in different countries. In our cohort, children receiving AOMs prescriptions demonstrated significantly higher BMI z-scores and a greater prevalence of obesity-related comorbidities compared to their counterparts not prescribed AOMs. This suggests that AOMs prescriptions may have been prioritized for individuals with more significant obesity. Similarly, Hidirsah et al. [15], in their investigation of pharmacotherapy use in a national cohort of 35,898 young patients with obesity, observed that higher BMI levels were significantly associated with an increased likelihood of being prescribed AOMs.

Our analysis revealed that the incidence of AOMs prescriptions was higher among individuals of Jewish ethnicity, those from higher SEP levels, and females. This pattern may be influenced by sociocultural beliefs regarding ideal body image, particularly within Jewish communities and higher SEP groups, as well as a greater acceptance of larger body sizes among individuals from Arab ethnic backgrounds and lower SEP levels. However, it remains unclear whether these disparities are driven by patient preferences, clinician bias, or limited access to healthcare providers comfortable with prescribing AOMs.

The observed sex disparity in AOMs prescriptions, which has also been reported in other studies focusing on pediatric populations [13–15], may reflect societal norms and ideals. Women are more likely than men to report experiences of weight-related stigma [21], which may contribute to a higher likelihood of seeking obesity treatment among females. Additionally, unconscious clinician bias may play a role, potentially leading to earlier initiation of AOMs therapy in females compared to males.

Furthermore, individuals with two or more AOMs purchases exhibited higher BMI z-scores, increased prevalence of T2D, hypertension, and NAFLD, as well as higher referral rates for dietitian consultations. These findings suggest greater adherence to medication treatment among those with a greater level of obesity or associated comorbidities.

In our real-world analysis, metformin emerged as the most frequently prescribed AOMs and was associated with the longest treatment duration. GLP-1 receptor agonist was the second most commonly prescribed AOMs, while orlistat and GLP-1 receptor agonist exhibited comparable treatment durations. Despite not being FDA-approved for the treatment of obesity, the preference for metformin may be attributed to its relatively favorable side effect profile, cost coverage, or patient and provider preference, given its widespread use for its primary indications.

Notably, 8.1% of individuals who purchased metformin also had a diagnosis of T2D. For these individuals, it is unclear whether the initial indication for metformin prescription was obesity or T2D. This dual indication may also explain the longer treatment duration observed for metformin compared to the other AOMs. The predominance of metformin prescriptions for obesity has also been reported by Czepiel et al. [14], who identified metformin as the most frequently prescribed AOMs among children and adolescents, followed by topiramate.

Our data indicates that the initiation of AOMs therapy (including both metformin and GLP-1 receptor agonist) was associated with a significant reduction in BMI z-scores, followed by a subsequent increase upon discontinuation of treatment, although the BMI z-scores remained lower than baseline levels. This pattern aligns with prior observations [22, 23]. While the weight reduction observed in our cohort was modest, existing studies have demonstrated that even a moderate reduction in weight, when combined with behavioral changes and adjunctive pharmacotherapy, can significantly mitigate health risks associated with obesity-related conditions [24]. In our study, of those treated with AOMs, 55.1% had a maximal BMI-z score reduction during treatment of >0.2 SD.

A systematic review conducted for the US Preventive Services Task Force (USPSTF), which evaluated recent randomized controlled trials (RCTs), found that control groups were more likely to continue gaining excess weight compared to those receiving pharmacologic treatment. The researchers concluded that even a stabilization of weight gain could be clinically significant over the long term [25].

In our study, we observed that the initiation of AOMs resulted in substantial reductions in blood glucose levels, HbA1c, triglycerides, and total cholesterol, accompanied by a significant increase in HDL cholesterol levels for both sexes.

After treatment cessation, there was a trend toward increases in blood glucose, HbA1c, triglycerides, and total cholesterol, although these values remained lower than pre-treatment levels. HDL cholesterol declined post-treatment but remained elevated compared to baseline values.

A prior systematic review [26], which included 24 RCTs with 1 623 participants aged 4–19 years and follow-up ranging from 2 months to 2 years, showed that metformin produced a modest reduction in BMI z-scores (mean change range: -0.37 to -0.03 vs. -0.22 to 0.15) and improved insulin resistance (mean change range: -3.74 to 1.00 vs. -1.40 to 2.66).

The absence of a significant reduction in BMI z-scores or improvements in laboratory parameters in participants treated with orlistat in our study may be attributed to the small sample size of those treated with orlistat or the limited impact of the medication itself. This finding is consistent with results from a systematic review and meta-analysis of adolescents with overweight and obesity, which reported no significant effects of orlistat on body weight, BMI, lipid profiles, or serum glucose levels [27].

A recent published systematic review and meta-analysis conducted of RCTs with <18-year-olds of pharmacotherapeutic agents published up to November 2022 examined data on cardiometabolic risk factors and anthropometry [28]. Overall, 35 RCTs were included. Trials examined metformin (*n* = 26), GLP1 receptor agonists (*n* = 7) and orlistat (*n* = 2). Intervention duration varied (3–24 months). Metformin had resulted in moderate reductions in triglycerides (mean difference −10.6, 95% CI − 18.6 to −1.8 mg/dL), a moderate decline in insulin resistance, and a small to moderate decline in BMI z-score (mean difference −0.26 95% CI − 0.39 to −0.14). Response to GLP1 receptor agonists was heterogeneous and results of subgroup analysis demonstrated variability of impact. Liraglutide (2 RCTs) resulted in a small reduction in HOMA-IR and BMI z-score (mean difference −0.15 95% CI − 0.25 to −0.06), but little to no benefit on other outcomes. Orlistat had a moderate reduction in diastolic blood pressure and little to no benefit in other outcomes measured.

The observed gap in the effectiveness of pharmacological interventions between real-world settings and RCTs can be attributed to several factors. Firstly, RCTs recruit participants through rigorous inclusion and exclusion criteria, resulting in a relatively homogeneous study population. Conversely, real-world settings involve a more diverse group of patients with varying demographic and medical characteristics, potentially affecting treatment outcomes.

Secondly, medication persistence is generally lower in real-world settings compared to RCTs. This difference may stem from variations in protocols, as RCTs often include more frequent interactions with multidisciplinary teams, fostering greater adherence to prescribed regimens. Moreover, treatment in RCTs is typically conducted at specialized centers with extensive experience, which may further enhance adherence rates.

Thirdly, lifestyle interventions, such as dietary and behavioral counseling, play a less prominent role in real-world settings. Patients in routine practice often have limited access to professional dietary guidance and structured behavioral therapy compared to the comprehensive support provided in RCTs.

Nevertheless, our study demonstrates that AOMs are effective in real-world settings, achieving significant weight reduction and improvements in metabolic parameters, even with relatively short treatment durations.

### Strengths and Limitations

Our study has several limitations. First, the relatively low persistence rates for treatment with AOMs in our cohort limited our ability to accurately assess the efficacy of these medications in the long-term. The real-world nature of the study may partially explain the low persistence, as a higher number of patients discontinued medications due to side effects, lack of efficacy, or medication costs compared to what is typically observed in RCTs. Second, we lacked data on adherence to lifestyle modifications, which are integral to the effectiveness of AOMs. However, according to clinical guidelines, AOMs are prescribed to pediatric patients who have already initiated lifestyle changes. Third, due to the retrospective and observational design of the study, we cannot definitively confirm that patients who purchased the medications actually took them. Nonetheless, as we utilized pharmacy claims data (which required a copayment), we assume that most patients who purchased the medications also used them. Fourth, our study did not capture medication dosing, which could influence the efficacy of the treatments. Lastly, our analysis did not include data on the use of weekly GLP-1 receptor agonist (semaglutide) which was approved for pediatric use in Israel only in 2024. Up to now, it is available exclusively through private purchase, and no available data on its utilization was accessible at the time of our study.

Despite these limitations, the strengths of our study include its relatively large sample size compared to previous real-world studies [13, 14]. We utilized the CHS database, the largest HMO in Israel, ensuring comprehensive inclusion of data on demographic characteristics, comorbidities, medication possession, and outcomes. Furthermore, to the best of our knowledge, our study is the first to evaluate prescribing patterns and usage of a variety of AOMs in the pediatric population in Israel.

## Conclusion

Our findings contribute to the growing body of research examining the efficacy of AOMs in real-world settings for the pediatric population, providing a better understanding of treatment effectiveness in everyday clinical practice. Our study suggests that AOMs may play a role in weight management for children and adolescents with obesity and should be considered a key component of a multimodal approach to managing this serious and harmful condition. However, the low persistence rate observed in our study may reduce the strength of our conclusions. Therefore, further studies are required to fully assess the effectiveness of FDA-approved AOMs for weight loss in pediatric patients in real-world settings.

## Data Availability

The datasets generated and analyzed during the current study are not publicly available due to the privacy policy of Clalit Health Services, but are available from the corresponding author on reasonable request, contingent upon approval from the legal department of Clalit Health services for the transfer of the datasets and the signing of a suitable appropriate Non- Disclosure Agreement.
